# Modeling cellular deformations using the level set formalism

**DOI:** 10.1186/1752-0509-2-68

**Published:** 2008-07-24

**Authors:** Liu Yang, Janet C Effler, Brett L Kutscher, Sarah E Sullivan, Douglas N Robinson, Pablo A Iglesias

**Affiliations:** 1 Electrical & Computer Engineering, Johns Hopkins University, Baltimore, MD 21218, USA; 2 Cell Biology, Johns Hopkins School of Medicine, Baltimore, MD 21205, USA

## Abstract

**Background:**

Many cellular processes involve substantial shape changes. Traditional simulations of these cell shape changes require that grids and boundaries be moved as the cell's shape evolves. Here we demonstrate that accurate cell shape changes can be recreated using level set methods (LSM), in which the cellular shape is defined implicitly, thereby eschewing the need for updating boundaries.

**Results:**

We obtain a viscoelastic model of *Dictyostelium *cells using micropipette aspiration and show how this viscoelastic model can be incorporated into LSM simulations to recreate the observed protrusion of cells into the micropipette faithfully. We also demonstrate the use of our techniques by simulating the cell shape changes elicited by the chemotactic response to an external chemoattractant gradient.

**Conclusion:**

Our results provide a simple but effective means of incorporating cellular deformations into mathematical simulations of cell signaling. Such methods will be useful for simulating important cellular events such as chemotaxis and cytokinesis.

## Background

Many cellular processes are characterized by substantial shape changes. For example, chemotaxing cells become polarized, assuming a highly elongated form, and crawl across solid substrates in the direction of increasing concentrations of chemoattractant [1]. During cytokinesis, a single cell undergoes significant cytoskeletal deformation, reforming into two daughter cells [2]. These cellular processes are fundamentally mechanical, utilizing force generation at the molecular scale to generate shape changes. Properly simulating cellular shape change requires that we have a description of the underlying mechanical properties of the cell.

To understand fully the mechanisms that regulate these cell shape changes requires knowledge of the signaling pathways as well as their effect on the mechanical properties of cells. For example, a complete model of chemotaxis would require a description of the gradient sensing capability of cells together with a physical model for the cellular migration [3]. Few such models exist, even though it is now appreciated that the response of cell-signaling pathways can be regulated in response to alterations in cell size and shape [4]. The traditional method of simulating cellular deformations is by specifying the boundary of the cell explicitly through a finite-element model (FEM) [5-7]. One problem is that simulation of biological shape deformations – which invariably involves solving partial differential equations on moving boundaries – can be computationally expensive particularly when the cellular deformations are not small. During many processes including cytokinesis and chemotaxis, cellular shape deformations tend to be large and occur rapidly. Here, we demonstrate how the Level Set Method (LSM) can be used to couple mechanical models of the cell with biochemical models of signaling pathways to simulate large cellular deformations.

We briefly contrast the LSM approach to other methods that have been used to account for cellular deformations.

The immersed boundary method (IBM), introduced by Peskin [8] was developed to simulate the interaction of flexible tissues with the surrounding incompressible fluid. It has been used to simulate cell shape changes during motility [9]. In the IBM, the Navier-Stokes equation describing the fluid flow can be solved on a fixed grid, simplifying this computationally expensive step. The membrane and cytoskeleton is discretized by assigning a series of nodes that are connected by viscoelastic elements. As the cell deforms, nodes and their corresponding links have to be inserted or deleted. This book-keeping comes at a considerable computational complexity. For this reason, the IBM may best be used in situations where the cell shape does not change considerable [10].

More recently, the cellular Potts model (CPM) has become a popular vehicle to simulate cell shape changes [11]. In the CPM, a cell is described by a connected domain of pixels on a regular grid. The shape of the cell is evolved by updating each pixel based on a set of probabilistic rules. This method does not use an explicit viscoelastic description of the cell. Instead, cell shape is constrained by minimizing an energy function that penalizes size-deformations as well as membrane bending. Cellular Potts models have been used to simulate two-dimensional (2-D) models of cell motility in fish keratocytes [12] and amoebae [13]. Unlike FEM or IBM, modeling large changes in the shape of the cell is no more computationally expensive than small changes. One drawback, however, is that the mechanical description of cells in the CPM framework is not as tightly integrated with experimentally-based measurements as the method presented here.

Models of cellular shape changes have all been derived based on explicit descriptions of the cell morphology that are updated based on the simulated behavior of the underlying cytoskeleton. For example, Rubinstein *et al*. provide a detailed 2-D computational model of the lamellipodium keratocyte motility [14]. In this model, the cellular domain is updated at each time step based on the protrusive and retractive forces (actin polymerization and acto-myosin contraction) and re-gridded. This avoids the necessity for nodes and keeping track of the mechanical state of the system. However, the model relies on an elastic (rather than viscoelastic) network which may be appropriate for the thin keratocyte, but is not likely to be applicable to thicker cells.

The rest of the paper is organized as follows. We first provide some necessary background. We then develop a mechanical model within the LSM that accounts for the viscoelastic nature of the cell. We fit this model to experimental data obtained through micropipette aspiration experiments on *Dictyostelium *cells. We incorporate this viscoelastic model into a level set framework and illustrate how large-scale shape deformations can be accounted by the model. This is done through simulations by showing that the model accurately captures the behavior of the aspirated cell. Finally, using a simple gradient sensing model to generate internal force profiles, we simulate the changing morphology of a cell chemotaxing in response to an externally applied chemoattractant gradient. Using the framework developed here, we obtain the force profiles needed to achieve stable migrating cell morphologies observed for several strains. The methods developed here allow us to link forces acting on the cell and mechanical properties of the cytoskeleton to cell shape deformation explicitly, and will prove useful in studying cellular processes undergoing large-scale shape changes.

### Biological background

Cells derive their mechanical properties from actin, actin-associated proteins, and motor proteins such as myosin-II [2], which are components of the cytoskeleton. Though distributed throughout the cell, the actin cytoskeleton is concentrated along the periphery of the cell underneath the membrane, particularly in *Dictyostelium*, and is the molecular machinery that generates cellular shape changes during cell division and chemotaxis.

Cytoskeletal networks exhibit viscoelastic behavior, having both viscous and elastic properties [15-17]. Actin filaments alone do not create significant mechanical resistance; instead, cross-linking of actin filaments by various actin binding proteins imparts mechanical rigidity to the cell. Under applied load, cross-linked actin networks behave similarly to an elastic solid and can be described using Hooke's law. However, because cross-linking proteins bind to and dissociate from actin filaments, actin-based networks may also exhibit viscous flow. Myosin-II, in filament form, also binds to actin filaments and provides mechanical resistance of the cell, as well as influencing the binding kinetics of various actin crosslinkers [2,18]. The interior of the cell also contains cytoskeletal polymers, as well as organelles, a nucleus, and cytoplasmic fluid. Thus, owing to their viscoelastic nature, cells exhibit a time-dependent deformation in response to mechanical force.

### Introduction to level set methods

**Figure 1:**
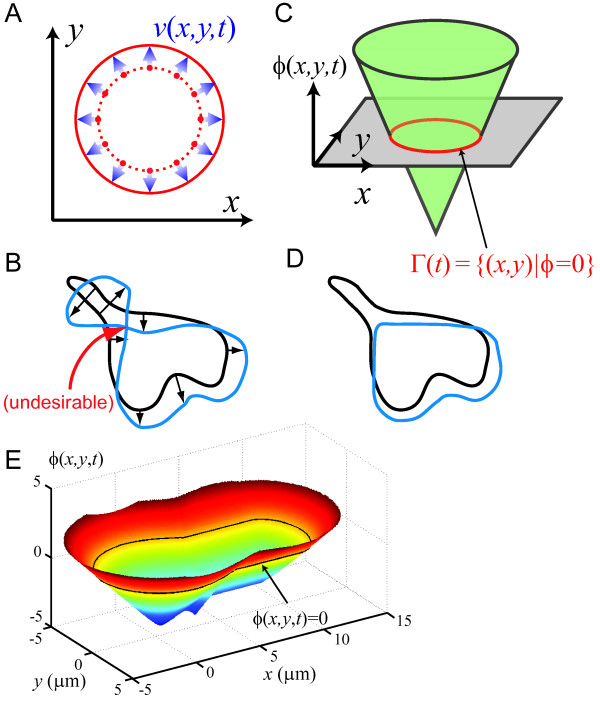
**Introduction to level set methods**. A. The traditional method of tracking moving boundaries involves discretization of the boundary (dotted red) into a set of points, moving each point **x **= (*x*, *y*) according to the local velocity (*v*(**x**, *t*)), leading to a new boundary at the new locations (solid red). B. Difficulties can arise, however, when the geometry of the boundary becomes irregular. In this case, the point tracking method often fails to preserve the boundary topology. Special attention is required to resolve these errors, increasing computational costs. C. In the Level Set Method (LSM), the boundary Γ(*t*) is embedded into a higher dimensional potential function (*ϕ*(**x**, *t*)) as the zero-contour. Γ(*t*)moves as *ϕ*(**x**, *t*)evolves in time. D. Because the boundary is defined implicitly, the LSM framework overcomes some of the difficulties of boundary point tracking. E. This example illustrates how an arbitrary cell shape (black contour) can be embedded into a signed distance function to form the level set potential function *ϕ*(**x**, *t*). In this case, the potential function is given by the Euclidean distance to the cell boundary, with positive (resp. negative) sign when outside (resp. inside) the cell.

Cell motion has been traditionally simulated by: discretizing the cell boundary, computing the displacement of each of the points according to the local velocity, and forming a new boundary with the displaced points (Fig. 1A). This method may run into difficulties when the spatial or temporal resolution of the simulations is not sufficiently fine, or when changes in topology occur (Fig. 1B). The Level Set Method (LSM) can be used to overcome these difficulties [19]. LSM is a numerical technique for tracking interfaces and shapes which has been widely used in various fields including computer graphics [20], image processing [21], computational fluid dynamics [22] and material science [23].

Suppose that the cell boundary at time *t *is described by the closed-contour Γ(*t*). The LSM requires a potential function (Fig. 1C.), denoted as *ϕ*(**x**, *t*), that is related to Γ(*t*) according to:

Γ(*t*) = {**x**|*ϕ*(**x**, *t*) = 0}.

Thus, Γ(*t*) is the *zero-level set *of *ϕ*(**x**, *t*). It follows that, in the LSM, the cell membrane is represented implicitly through the potential function which is defined on a fixed Cartesian grid, thus eliminating the need to parameterize the boundary. This allows the LSM to handle complex boundary geometries efficiently (Fig. 1D).

One candidate for the potential function is the signed distance function [24], defined by:

(1) $$\text{signd}(x,\Gamma )=\{\begin{array}{cc} \begin{array}{c} -d(x,\Gamma ),\\ d(x,\Gamma ),\\ 0, \end{array} & \begin{array}{l} \text{if }x\in S,\\ \text{if }x\notin S,\\ \text{if }x\in \Gamma , \end{array} \end{array}$$

where *S *identifies the area occupied by the cell and *d*(**x**, Γ) is the distance of position **x **to the curve Γ; see Fig. 1E for an example of a cell shape embedded in a potential function derived from the signed distance function.

We now manipulate Γ(*t*) implicitly through the function *ϕ*(**x**, *t*) according to the equation:

(2) $$\frac{\partial \phi (x,t)}{\partial t}+v(x,t)\cdot \nabla \phi (x,t)=0.$$

The vector **v**(**x**, *t*) is the velocity of the level set moving in the outward normal direction. In our case, **v**(**x**, *t*) intrinsically describes the cell's membrane protrusion and retraction velocities. These velocities can be driven by externally applied forces on the cell membrane (e.g. from a micropipette aspirator), or internally generated mechanical forces (e.g. actin polymerization or myosin-II retraction), or both. To determine how these forces translate to membrane velocity, however, first requires a mechanical model of the cell.

As the potential function corresponding to the cell shape is evolved, it can become quite steep or flat. To reduce the numerical errors caused by these effects, we re-initialize the potential function periodically [24]. This can be done using the re-initialization equation [25]:

$$\frac{\partial \phi (x,t)}{\partial t}=S(\phi (x,0))(|\nabla \phi (x,t)|-1),$$

where *S*(*ϕ*(**x**, 0)) is taken as +1 inside the cell, -1 outside the cell and zero on the cell membrane.

## Results and Discussion

### Viscoelastic model of cell deformation

The LSM relies on a continuum description of the material properties of the cell [2,26]. We use mechanical models to describe the viscoelastic behavior of the cell [27]. Our mechanical model is based on a representation of cells that assumes a viscoelastic cortex surrounding a viscous core. For cells where intracellular components, such as the nucleus, take a considerable fraction of the cellular volume and play an active role in determining cell shape, the method described here will not be applicable without explicitly modeling these internal structures.

**Figure 2:**
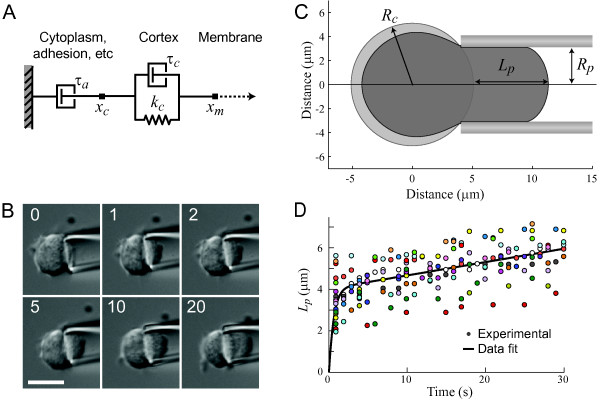
**Viscoelastic model of cell**. A. Representation of the viscoelastic model of the cell. *x*_*c *_and *x*_*m *_denote the location of the cell cytoplasm and membrane, respectively; *τ*_*c *_and *k*_*c *_define the mechanical model of the cell cortex; *τ*_*a *_includes the viscous deformation of the cytoplasm as well as other components including adhesion. B. To validate our model and determine model parameters, we utilized micropipette aspiration technique. Relevant parameters include the radius of the micropipette (*R*_*p*_), the radius of the cell (*R*_*c*_), and the length of protrusion into the micropipette (*L*_*p*_). C. Example of a *Dictyostelium *cell being aspirated into the micropipette at 0.65 nN/*μ*m^2^. Time stamps are in seconds, scale bar shows 10 *μ*m. D. Protrusion into the pipette was measured and was accounted for by the model. Different colored circles represent data from 22 individual experiments. Solid line represents the deformation defined by Eqn. 6 with parameters *k*_*c *_= 0.098 nN/*μ*m^3^, *τ*_*c *_= 0.064 nNs/*μ*m^3 ^and *τ*_*a *_= 6.09 nNs/*μ*m^3^.

We model the cortex connecting the cell membrane and the cytoplasm with a Voigt model, which consists of the parallel connection of an elastic element *k*_*c *_(nN/*μ*m^3^) and a viscous element *τ*_*c *_(nNs/*μ*m^3^). The latter describes the association and dissociation dynamics of the cross-linkers. We model the cytoplasm by a purely viscous element, *τ*_*a *_(nNs/*μ*m^3^), which is placed in series with the Voigt model (Fig. 2A). The element *τ*_*a *_includes contributions from the interior of the cell as well as adhesion, friction and cytoskeletal reorganization. Strains of the cortex and cytoplasm are described by the variables *x*_*m *_and *x*_*c*_, respectively. Note that, in our simulations, we use pressure rather than force to induce the cellular deformations; this accounts for the extra *μ*m^2 ^found in the denominators of the parameters in our model.

As shown below, this combined Voigt-dashpot viscoelastic model reasonably approximates the mechanical properties of *Dictyostelium *where cross-linking proteins are predominantly enriched in the cortex. Extending our framework to other cell types may require different viscoelastic models to describe the cell of interest. For example, aspiration of chondrocytes suggests that these cells obey a Kelvin model (similar to the Voigt element, but includes an elastic component in series with the viscous element) [28]. Once the appropriate viscoelastic model is developed, the implementation in the LSM framework introduced here is straightforward.

#### Experimental determination of model parameters

To determine appropriate parameters for the viscoelastic model, we used micropipette aspiration to apply step pressures (rapid increase of pressure from 0 to 0.65 nN/*μ*m^2^) to individual cells [29,30]. In this technique, a small negative hydrostatic pressure is created at the tip of a micropipette. By bringing the micropipette into close proximity of the cellular surface, the cell is aspirated into the micropipette.

We applied step pressures to wild type interphase cells and measured cellular deformation as a function of time (Fig. 2B). Deformation is quantified by the length of cellular protrusion into the pipette tip, denoted *L*_*p *_(Fig. 2C). We aspirated 22 cells with a radius of 4.3–6.1 *μ*m, a pipette radius of 3.1 *μ*m and a pressure of 0.65 nN/*μ*m^2^. The cells deformed in two distinct phases (Fig. 2D). Within the first several seconds after application of the aspirator, the cellular deformation increased sharply, with the length of the aspirated cortex increasing to an average value of 4 *μ*m. The deformation during this phase can be interpreted as being dominated by the elastic characteristics of the cytoskeletal network. Thereafter, the trajectory was dominated by slow cellular flow into the micropipette, increasing, on average, about 2 *μ*m over the next 25 s.

The pressure applied by the micropipette aspirator is not the only pressure experienced by the cell. At rest, the cell is also under pressure from cortical tension (*γ*_*ten*_), which maintains the spherical shape of the cell. Under the cortical shell-liquid drop model [31], we assume that the cortical tension arises as surface tension (ignoring tangential stress). Following the Young-Laplace equation for liquid interfaces, the equilibrium pressure experienced by a spherical cell of radius *R*_*c *_is

(3) *P*_eq _= 2*γ*_ten_/*R*_*c*_.

The cell's protrusion into the micropipette is driven by the aspiration pressure. As the cell is aspirated, the portion of the cell inside the micropipette will be a spherical cap of radius *R*_cap _<*R*_*c*_. Given a measured length of protrusion *L*_*p*_, the radius of the spherical cap *R*_cap _can be obtained (see Additional file 1). The cap's smaller radius gives rise to higher local curvature, creating a rounding pressure:

*P*_round_(*L*_*P*_) = 2*γ*_ten_/*R*_cap _- *P*_eq_,

to oppose the aspiration

At the critical aspiration pressure, *P*_crit_, the cell extends a perfect hemispherical projection with radius *R*_*p *_into the micropipette and does not protrude any further under this constant pressure. Thus, the critical pressure is:

(4) *P*_crit _= *P*_round_(*R*_*p*_) = 2*γ*_ten_(1/*R*_*p *_- 1/*R*_*c*_).

The cortical tension has been measured to be 1–1.5 nN/*μ*m in passive, wild type *Dictyostelium *cells [18,31,32]. Here, assuming cortical tension of *γ*_ten _= 1 nN/*μ*m, pipette radius of *R*_*p *_= 3.1 *μ*m and cell radius of *R*_*c *_= 5.1 *μ*m, we can compute *P*_crit _to be approximately 0.25 nN/*μ*m^2^. Because the applied pressure was greater than the critical pressure, the cell was continuously aspirated into the pipette. Cells were only tracked for 30 s, as longer timescales are dominated by cortical remodeling and turnover [33,34].

Pascal's law dictates that the hydrostatic pressure, *P*_ext_, in the micropipette is normal to the cell membrane inside the micropipette and has the same magnitude in all directions. Similarly, the cell's equilibrium pressure is normal to the cell membrane everywhere with the same magnitude. We used the total pressure, *P*_total _= *P*_ext _- *P*_round_, as the input to the cell's mechanical model. This pressure is applied to the cell membrane region around *x*_*m *_and is transferred directly to the cell's cortex, formed of cytoskeleton and its cross-linkers, just beneath the cell membrane. The corresponding mathematical model is:

$$\begin{array}{l} P_{\text{total}}=\tau_{c}({\dot{x}}_{m}-{\dot{x}}_{c})+k_{c}(x_{m}-x_{c}-w_{0})\\ P_{\text{total}}=\tau_{a}{\dot{x}}_{c}, \end{array}$$

where *w*_0 _represents the initial position of the cell cortex when no force is applied to the system. We define ℓ such that:

*x*_*c *_= *x*_*m *_- ℓ - *w*_0_.

With this variable change, the transformed system can be written as:

(5) $$\left[\begin{array}{c} {\dot{x}}_{m}\\ \dot{\ell } \end{array}\right]=\left[\begin{array}{cc} 0 & -k_{c}/\tau_{c}\\ 0 & -k_{c}/\tau_{c} \end{array}\right]\left[\begin{array}{c} x_{m}\\ \ell \end{array}\right]+\left[\begin{array}{c} 1/\tau_{a}+1/\tau_{c}\\ 1/\tau_{c} \end{array}\right]P_{\text{total}}.$$

Using the Voigt-dashpot model of Eqn. 5 to account for the viscoelastic response of the cell to an applied step pressure, the aspirated cellular length into the pipette, *L*_*p*_, is given by:

(6) $$L_{p}(t)=x_{m}(t)=P_{\text{total}}\left(\frac{1}{k_{c}}(1-e^{-k_{c}t/\tau_{c}})+\frac{t}{\tau_{a}}\right).$$

Data from all 22 cells were combined. The following parameters in the viscoelastic model were obtained using a least squares fit (using Matlab's curve fitting toolbox): *k*_*c *_= 0.098 ± 0.007 nN/*μ*m^3^, *τ*_*c *_= 0.064 ± 0.018 nNs/*μ*m^3^, and *τ*_*a *_= 6.09 ± 1.44 nNs/*μ*m^3 ^(the ± value refer to a 95% confidence interval). With these parameter values, the 1-D model was able to capture the deformation trends observed in the experimental data (Fig. 2D). Note that the elastic constant obtained, when applying the methods of Theret *et al*. [35] and Hochmuth [30], is equivalent to an elastic Young's modulus of 70 pN/*μ*m^2^, which is similar to the value of 95 pN/*μ*m^2 ^measured for *Dictyostelium *using different techniques [18].

### Implementation of micropipette aspiration simulation

During micropipette aspiration, the cell's velocity is generated by externally applied pressure, as well as internally generated cellular pressures such as cortical tension. We now outline how the contribution of each pressure is computed and applied to the cell's potential function *ϕ*(**x**, *t*).

We choose to do the simulations in two dimensions. The level set method is directly applicable to three dimensions (3-D), and all of the level set equations either carry over without change into 3-D, or have natural extensions. In practice, however, the computational burden of 3-D simulations is significant and hence we restrict ourselves to two dimensions. To differentiate the forces (and hence pressures) which are 2-D, from the scalar pressures used above, we use bold characters.

#### Evolving the cell membrane

The simulation accounts for the effects of three pressures: those generated externally by the micropipette; those generated internally to maintain constant volume; and rounding pressure corresponding to the cell's cortical tension. Together, these pressures generate a velocity field that evolves the cell's membrane.

##### Externally applied pressure

To account for the force generated by the hydrostatic pressure in the micropipette, the pressure **P**_ext_, uniform in magnitude and normal to the cell membrane, is used in the LSM simulation. This force exists only inside the inner boundaries of the pipette.

##### Pressure due to volume conservation

We assume that, during aspiration, the cellular volume (*V*) remains constant. To enforce this constant volume condition numerically, we implement a pressure, acting normal to the surface:

(7) **P**_vol _= *K*_vol_(*V*_resting _- *V*_actual_)**n**

where **n **is the outward normal. The cell's volume is evaluated by assuming the cell is radially symmetric:

(8) *V*_actual _= ∫_cell length_*πr*(*x*)^2^*dx*.

**Figure 3:**
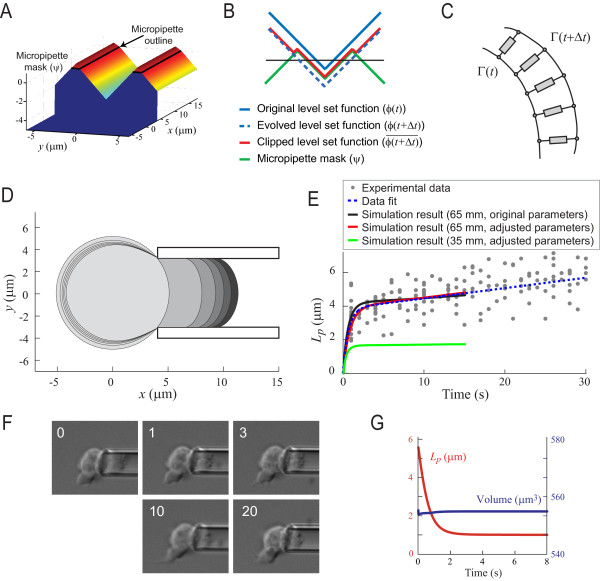
**Simulation of micropipette aspiration**. A. To account for the solid surface of the micropipette, we introduce a mask potential function (Ψ) defined by the micropipette walls (black line). B. A cross section illustrates how the masking potential function is used to clip the evolving level set potential function. Based on the driving equations, the potential function evolves from *ϕ*(*t*)(solid blue) to *ϕ*(*t *+ Δ*t*)(dashed blue). However, this new position makes the cell cross into the pipette (defined by the mask function Ψ – green line). The level set function is then clipped to $\bar{\phi (t+\Delta t)}$ to account for the solid surface. C. Parallel spring-dashpot units are used to represent the viscoelastic state of the cell as the boundary evolves from Γ(*t*) to Γ(*t *+ Δ*t*). Each component consists of a viscoelastic model as defined in Fig. 2A. D. Simulation of micropipette aspiration implementing the viscoelastic model of the cell in the LSM (using the adjusted parameters; see main text). Shown is an overlay of simulation frames at *t *= 0 s (spherical cell, light grey), 0.5 s, 1 s, 5 s and 20 s (farthest protrusion, black). E. Measurements from aspiration simulations. Assuming an aspiration pressure of 0.65 nN/*μ*m^2^, the protrusion into the cell from the simulation (black line) can account for the experimental data (grey dots; mean square error, MSE, is 0.74 *μ*m; coefficient of determination, *R*^2^, of 0.78) nearly as well as the data fit (dotted line) from Fig. 2D. (MSE: 0.73, *R*^2^: 0.79). With slightly different parameters (see main text) the simulation (red line) overlaps the fitted data better (MSE: 0.73, *R*^2^: 0.79). Aspiration forces near the critical pressure (0.35 nN/*μ*m^2^) can deform the cell initially, but do not draw it in further (green line). F. After 20 s of micropipette aspiration, the pressure in the aspirator is dropped, leading to a relaxation in the protrusion distance; a typical example is shown here. Time stamp indicate seconds after release of aspiration pressure, scale bar corresponds to10 *μ*m. Note that the cell does not fully retract the aspirated portion. G. In a LSM simulation, the cell's retraction can be shown as the decrease of length of protrusion. Simulation of cell relaxation accurately demonstrates the lack of complete retraction observed (red). Also shown is the cell's volume (blue) during the simulation demonstrating that any volume changes are minimal.

To ensure that the cell's volume does not change during the course of the aspiration requires that *K*_vol _be large. In our simulations, we set *K*_vol _= 0.1 nN/*μ*m^5^, which was sufficiently high to ensure that volume changes were minimal (Fig. 3G) while maintaining stability of the simulations.

##### Rounding pressure due to cortical tension

Resting cells experience cortical tension [31] which generates pressure, **P**_eq_, as shown in Eqn. 3. When a spherical cell is aspirated, the cell's cortex resists deformation.

The pressure generated depends on the local surface curvature

$$\kappa (x)=\nabla \cdot \left(\frac{\nabla \phi }{\left|\nabla \phi \right|}\right),$$

and a material property of the cortex referred to as the cortical tension (*γ*_ten_) according to:

(9) **P**_ten_(*x*) = 2*γ*_ten_*κ*(*x*)**n**.

Therefore the rounding pressure produce by the cell is **P**_round _= **P**_ten _- **P**_eq_. This acts inward normal to the membrane.

We have chosen to define **P**_round _as the difference between the tension and an equilibrium pressure. This is accordance to experiments on neutrophils that found that cortical tension depends on surface area [36]. However, the latter term can also be incorporated into the volume conservation term. In particular, combining **P**_vol _and **P**_round _leads to:

$$\begin{array}{c} P_{\text{vol}}-P_{\text{round}}=K_{\text{vol}}(V_{\text{resting}}-V_{\text{actual}})n-(2\gamma_{\text{ten}}\kappa (x)n-2\gamma_{\text{ten}}/R_{c})n\\ =K_{\text{vol}}(V_{\text{resting}}-{\hat{V}}_{\text{actual}})n-(2\gamma_{\text{ten}}\kappa (x))n, \end{array}$$

where ${\hat{V}}_{\text{actual}}$ = *V*_actual _+ 2*γ*_ten_/(*R*_*c*_*K*_vol_).

The coefficient 2 in the pressure equation is introduced to account for the fact that our curvature calculation is based on the 2-D representation of cell shape, as the curvature of a sphere of radius *r *is 2/*r*, but the curvature of a 2-D circle is only 1/*r*. In the computation of curvature, spline-based interpolation was used to smooth out discretization noise.

##### Total pressure and cell evolution

In the above formulations, the total pressure outward normal to the cell membrane is:

(10) **P**_total _= **P**_ext _+ **P**_vol _- **P**_round_.

The formulation of ${\dot{x}}_{m}$ in Eqn. 5 provides us with the pressure-velocity relationship:

(11) $$v={\dot{x}}_{m}=-(k_{c}/\tau_{c})l+(1/\tau_{a}+1/\tau_{c})P_{\text{total}}.$$

The velocity vector, **v**, is defined for points on the cell membrane. This needs to be extrapolated to a velocity field to evolve the potential function *ϕ*. It is only the velocity variations tangential to a given interface that dictate the interface motion [37]. A velocity field that minimizes the normal component of the field variation is achieved by extrapolating the membrane velocity with the nearest neighbor method. In other words, the velocity **v**(**x**) at a point **x **can be set equal to the membrane velocity **v**($\bar{x}$) at the membrane location $\bar{x}$ closest to the point **x**. It has been shown that a signed distance function tends to stay a signed distance function when the closest neighbor extrapolation method is used [38]. We can now use this velocity field to evolve the cell membrane according to Eqn. 2.

Eqn. 11 points to a difference between the LSM model of cellular deformation and the one-dimensional (1-D), scalar model used to obtain the viscoelastic parameters (Eqn. 6). In the latter, the pressure is co-aligned with the direction of the viscoelastic components, implying that the direction of motion is also always inline with the direction of the applied pressure. In the LSM simulation, the pressure is applied normal to the cell membrane, but the viscoelastic component, **l**, does not have to have the same directionality, and the resultant velocity vector is not always normal to the cell membrane. While providing us with good starting point for the parameter estimation, the 1-D formulation therefore can not be expected to explain the 2-D simulation completely.

#### Restricting cell shape inside micropipette

As the cell's level set potential function moves into the micropipette, its shape is restricted to remain inside the micropipette. This is achieved by first defining a mask potential function [39], Ψ, for the micropipette (Fig. 3A). The effect of the mask is to correct for the cell's potential function by clipping it (Fig. 3B) according to:

$$\bar{\phi (t+\Delta t)}=\min(\phi (t+\Delta t),\psi ).$$

This restriction guarantees that the cell boundary never moves outside of the inner walls of the virtual micropipette. After this restriction, the net change in *ϕ *is: $\bar{\phi (t+\Delta t)}$ - *ϕ*(*t*), which translates (see Additional file 1) to an effective velocity that is normal to the cell membrane:

(12) $$\bar{v}=-\frac{\bar{\phi (t+\Delta t)}-\phi (t)}{\Delta t}\frac{\nabla \phi }{|\nabla \phi {|}^{2}}.$$

Thus, wherever clipping by the micropipette mask occurs, we must use this effective velocity to evolve the potential functions in simulation.

#### Evolution of the viscoelastic state of the cell

In our simulations, the cell can be represented by a series of parallel viscoelastic systems with the same parameters (Fig. 3C). These sub-systems are not interconnected, and the applied pressure on each system, *P*_total _as defined in Eqn. 10, is normal to the cell membrane. We argue that applying total pressure to the parallel unconnected spring damper systems used in this model closely approximates cellular behavior when the following conditions are met:

1. Membrane pressure profile is piecewise smooth. This is a reasonable assumption as, in practice, pressure profiles are piecewise smooth. Even when a point force is applied to a particular location of the cell membrane, membrane elasticity will diffuse this force and make the pressure smooth locally.

2. Simulation grid density is dense enough for simulation stability, but not much denser than the discretization of the membrane pressure profile. With this assumption, the interpolation nature of level set method acts like a low pass filter, where effects of artificial abrupt jumps in the pressure profile are smoothed.

Let **l**(**x**, *t*), **x **∈ Γ(*t*) be the viscoelastic state of the cell at time *t *and at a position **x **on the membrane. That is, |**l**| represents the length of the numerous parallel unconnected spring-damper systems. At a given position, **x**, on the membrane, there is a vector with length given by |**l**(**x**)| = |ℓ| in Eqn. 5, representing the state of a *single *spring-damper system. Then

$$\frac{dl}{dt}=\frac{\partial l}{\partial x}\frac{\partial x}{\partial t}+\frac{\partial l}{\partial y}\frac{\partial y}{\partial t}+\frac{\partial l}{\partial t}=[Dl]v+\frac{\partial l}{\partial t},$$

where *D *is the Jacobian operator, [*D***l**]**v **represents the displacement of the whole cell membrane, and $\frac{\partial l}{\partial t}=\dot{\ell }n$ as defined in Eqn. 5. The equation describing the evolution of **l **is:

(13) $$-\frac{k_{c}}{\tau_{c}}l+\frac{1}{\tau_{c}}P_{\text{total}}=[Dl]v+\frac{\partial l}{\partial t}.$$

#### Testing of model: Micropipette aspiration simulation

**Figure 4:**
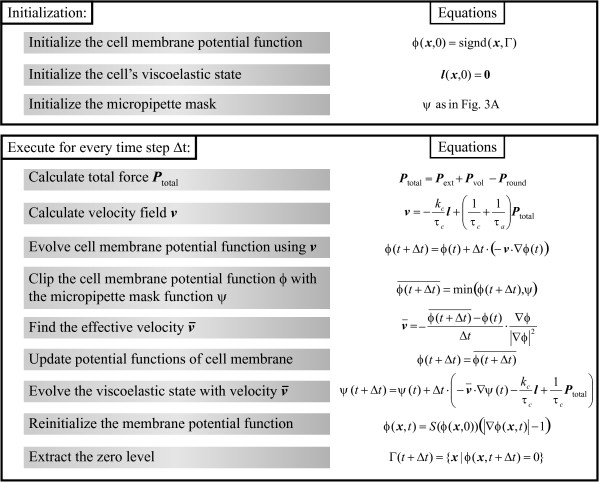
Algorithm for LSM simulation of micropipette aspiration.

To summarize, the flow chart of the simulation steps is shown in Fig. 4. The implementation is derived from the Level Set Toolbox [39] and is coded in Matlab (Mathworks, Natick, MA). The simulations were implemented on a fixed grid of 10 *μ*m in size, with density of 20 points/*μ*m and 4 ms time steps. Simulating 15 seconds of aspiration takes approximately 8 h on a desk-top computer.

We simulated the micropipette aspiration experiment under several different aspiration pressures. Using an aspiration pressure of 0.65 nN/*μ*m^2 ^(the pressure used to obtain our viscoelastic model parameters), our simulation reproduced the trend observed in real cells (black line in Fig. 3E). The result of this simulation did not completely overlap the least-squares fitted data, though the fit to the experimental data is nearly as good. The fitted data has a mean square error (MSE) of 0.73 *μ*m and a coefficient of determination (*R*^2^) of 0.79; the simulation has 0.74 *μ*m and 0.78 respectively. Using different parameter values: *k*_*c *_= 0.1 nN/*μ*m^3^, *τ*_*c *_= 0.08 nNs/*μ*m^3^, and *τ*_*a *_= 4.6 nNs/*μ*m^3^, we were able to reproduce the fitted data slightly more accurately (Fig. 3D and red line in Fig. 3E; MSE of 0.73 *μ*m and an *R*^2 ^value of 0.79).

Using 0.35 nN/*μ*m^2 ^of pressure, the cell was rapidly and partially aspirated into the pipette. Thereafter, it remained nearly immobile. This simulation recreates the observed behavior of *Dictyostelium *cells at aspiration pressures near the critical pressure.

To test our model further, we simulated the relaxation of an aspirated cell and compared this to experimental results in which a cell is aspirated into the micropipette for approximately 20 s at which point the applied pressure is released. The cell responds by rapidly retracting the aspirated portion (Fig. 3F). The retraction gradually slows to a near halt, with a significant portion of the cell remaining inside the micropipette. This behavior was reproduced in our simulations. The simulated cell retraction from the micropipette is measured in the reduction of length of protrusion (Fig. 3G), matching the retraction behavior seen in live cells. As shown in Fig. 3G, the variation in cell volume during these simulations was less than 1%.

### Simulating Dictyostelium cell shape changes using a simplified chemotaxis model

Having established that we can recreate the cellular shape during micropipette aspiration, in which externally applied pressures are driving cell shape changes, we consider a situation in which the pressures arise as a response to external stimuli. To this end we simulated the cell shape behavior of chemotactic *Dictyostelium *cells.

*Dictyostelium *cells have the ability to detect spatial differences in the concentration of the extracellular chemoattractant cAMP. They interpret these spatial differences and respond by localizing signaling molecules. These signaling molecules in turn bias the locations of actin polymerization driven protrusions and myosin-II motor mediated retractions, generating internal mechanical forces to deform the cell as well as propel the cell towards the chemoattractant [1,40].

Our goal in these simulations is not to propose new chemotaxis signaling mechanisms, or even to analyze the large number of proposed mechanisms (reviewed in [3]). Rather, it is to illustrate how cellular signaling can be coupled to the LSM framework to drive cellular deformations. Thus, we purposely implement a simple model connecting chemoattractant gradients with intracellular markers.

#### Implementation and testing

We base our model for pressure generation on a previously published signaling model that accounts for receptor mediated localization of phosphatidylinositol (3,4,5)-trisphosphate (PI(3,4,5)P_3_) [41]. Though recent experimental data suggests that cells employ multiple parallel pathways to regulate chemotaxis [42,43], localization of this membrane lipid has been correlated with the appearance of pseudopods [40]. Moreover, elevated levels of PI(3,4,5)P_3 _correlate temporally with increased levels of actin polymerization [44].

Rather than implementing the complete reaction-diffusion equations describing the PI(3,4,5)P_3 _model, we simplify it by using a steady-state distribution of PI(3,4,5)P_3 _along the cellular membrane. It was shown that the membrane concentration of PI(3,4,5)P_3 _is an amplified response of the relative cAMP concentration observed on the membrane [41,45]:

(14) PI(3,4,5)P_3 _∝ [cAMP/mean(cAMP)]^3^.

Next, we compute the pressure components contributing to cell motion, which include protrusion, retraction, volume conservation, and cortical tension pressures. To compute protrusion pressure, we first assume that actin polymerization creates a pressure wherever the PI(3,4,5)P_3 _concentration is above its mean level:

(15) $$P_{\text{prot}}=K_{\text{prot}}\max\left(0,\frac{\text{PI}(3,4,5){\text{P}}_{3}-\text{mean}(\text{PI}(3,4,5){\text{P}}_{3})}{\max(\text{PI}(3,4,5){\text{P}}_{3})-\text{mean}(\text{PI}(3,4,5){\text{P}}_{3})}\right)n.$$

Similarly, we assume myosin-II retraction occurs wherever PI(3,4,5)P_3 _concentration is below its mean level:

(16) $$P_{\text{retr}}=-K_{\text{retr}}\max\left(0,\frac{\text{mean}(\text{PI}(3,4,5){\text{P}}_{3})-\text{PI}(3,4,5){\text{P}}_{3}}{\text{mean}(\text{PI}(3,4,5){\text{P}}_{3})-\max(\text{PI}(3,4,5){\text{P}}_{3})}\right)n.$$

Both of these act normal to the cell membrane. We let the proportionality constant in Eqn. 14 be absorbed into constants *K*_prot _and *K*_retr_. Eukaryotic cells can generate actin mediated protrusion pressures of 0.5–5 nN/*μ*m^2 ^[46]. We chose *K*_prot _= 0.5 nN/*μ*m^2 ^and *K*_retr _= 1 nN/*μ*m^2^.

When computing the conservation of volume pressure, we assume that the cell is flat with uniform thickness. Thus, volume conservation is equivalent to conserving the 2-D area of the cell:

**P**_area _= *K*_area_(*A*_0 _- *A*)**n**.

The flat cell assumption also implies that the pressure generated by cortical tension depends only on the 2-D local surface curvature and the 2-D equilibrium pressure. The rounding pressure due to cortical tension is therefore given by:

(17) **P**_ten _= *K*_ten_(*κ*(*x*) - 1/*R*_*c*_)**n**.

Values of *K*_area _= 0.2 nN/*μ*m^4 ^and *K*_ten _= 1 nN/*μ*m were used in these simulations.

Summing all these components yields the total force normal to the cell membrane:

**P**_total _= **P**_prot _+ **P**_retr _+ **P**_area _- **P**_ten_.

**Figure 5:**
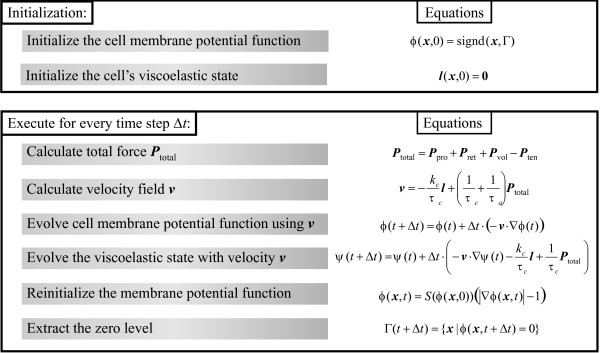
**Algorithm for LSM simulation of cell shape changes in response to external chemotaxis gradients**. This algorithm includes only general steps required to generate the pressure profile. To simulate chemotaxis also requires that the chemoattractant gradient be generated and that the protrusive (**P**_prot_) and retractive (**P**_retr_) pressures be computed. These would be determined by specific models of chemotactic response. In our simulations, these were generated by Eqn. 15 and Eqn. 16, respectively.

Finally, the membrane velocity is computed using Eqn. 11, with the same viscoelastic parameters *τ*_*a*_, *k*_*c *_and *τ*_*c*_. The simulation algorithm is similar to the micropipette aspiration case, and is summarized in Fig. 5.

**Figure 6:**
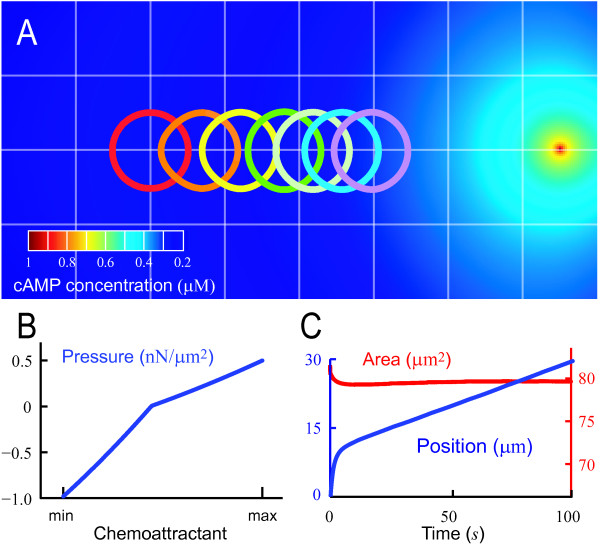
**LSM simulations of cell shape changes during chemotaxis**. A. We simulated the change in cellular morphology of a *Dictyostelium *cell exposed to a point source of chemoattractant (1 *μ*M of cAMP). Shown is the resultant chemoattractant field (computed by solving the diffusion equation) as well as the location of the cell at times 0, 1.5, 10, 40, 60, 80 and 100 s. Initially, the cell is assumed to be round (red circle). B. In this model, pressure was determined by the concentration of PI(3,4,5)P_3 _on the membrane as described in the text. The maximum and minimum refer to the concentrations experienced by the cell around the membrane. C. The position of the cell (blue) was plotted as a function of time, showing fairly constant velocity (11.7 *μ*m/min). Also shown is the cell's area (red) during the simulation demonstrating that changes are also minimal.

This simulation successfully generated chemotaxis behavior (Fig. 6). In response to a chemoattractant gradient, the cell, whose shape was initialized as a circle, changed shape and migrated in the direction of the chemoattractant gradient (Fig. 6A). The pressure profile (Fig. 6B) and displacement (Fig. 6C) are shown as functions of local cAMP concentration and time, respectively. The cell achieved a velocity of 11.7 *μ*m/min, which is similar to published velocities of *Dictyostelium *cells (e.g. 11.8 *μ*m/min[47]). During the simulation, the cellular area (and hence volume) remained nearly constant (Fig. 6C).

#### Membrane pressure profile and cell shape

**Figure 7:**
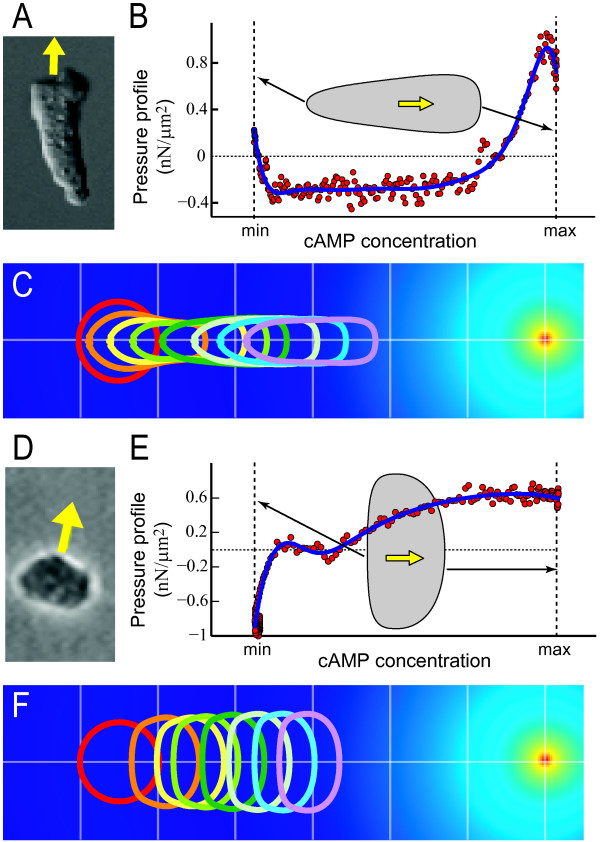
**Pressure profile drives cell shape**. A. During chemotaxis, wild type *Dictyostelium *cells acquire a polarized, elongated morphology. B. Eqn. 19 was used to compute the pressure profile (red dots) necessary to maintain the elongated cell shape (inset) along the cell membrane, and this is plotted as a function of the local chemoattractant (cAMP) concentration. The maximum and minimum refer to the concentrations experienced by the cell around the membrane. Pressure profile used in the simulations (blue line) was obtained by fitting the computed pressure profile, details are given in the Additional file 1. C. Chemotaxing cell using the pressure profile of panel B. The shapes of the cell are shown at times 0, 1.5, 10, 20, 40, 60, 80 and 100 s. Other details in the simulation are as in Fig. 6. D-F. Simulations of chemotaxis in *Dictyostelium amiB*^- ^cells. These mutant cells acquire a fan-like morphology (panel D) and move along their broad axis. This form of movement was recreated using the pressure profile of panel E (colors as in panel B). F. Chemotaxing cell using the force profile of panel E. Times of the shapes are as in panel C.

While our simulations of *Dictyostelium *recreate the motion of the cell in response to the chemoattractant gradient, the resultant cell shape change is small and the steady-state morphology does not resemble that observed experimentally in chemotaxing. Wild type chemotaxing *Dictyostelium *cells become elongated (Fig. 7A). Other strains, including the *amiB*^- ^mutants [48] can move stably in fan-like shapes that are reminiscent of keratocytes (Fig. 7D). Without determining the underlining molecular methods, we hypothesized that the difference in cell shape can be accounted for by the way that the force generation is distributed along the cell membrane. Our LSM simulation framework allows us to determine how these forces are distributed along the cell to generate the resulting cell shapes, both for wild type and mutants. To this end, we set out to replace our initial model, described by Eqn. 15 and 16, by one based on the observed morphologies.

Given a stable cell shape Γ_0 _traveling at velocity **u**, we let Γ_*u *_be the displaced cell at time Δ*t*, and *ϕ*_0 _and *ϕ*_*u *_be the potential functions representing Γ_0 _and Γ_*u *_respectively. The effective velocity field necessary for this displacement is:

(18) $$\bar{u}=-\frac{(\phi_{u}-\phi_{0})/\Delta t}{\left|\nabla \phi \right|}n.$$

If the cell shape is at steady state, we can assume that the internal viscoelastic network is also in steady state, that is, $\frac{dl}{dt}=0$. Therefore, from Eqn. 5, we compute the viscoelastic steady state ℓ = *P*_total_/*k*_*c*_.

Moreover, the membrane speed at steady state is expressed as ${\dot{x}}_{m}$ = *P*_total_/*τ*_*a*_. Combined with Eqn. 18, we find **P**_total_:

$$P_{\text{total}}=\tau_{a}\frac{(\phi_{u}-\phi_{0})/\Delta t}{\left|\nabla \phi \right|}n$$

Taking into account the effect of cortex tension, and assuming that there is no cell volume deviations, we can compute:

(19) $$P_{\text{prot}}+P_{\text{retr}}=\tau_{a}\frac{(\phi_{u}-\phi_{0})/\Delta t}{\left|\nabla \phi \right|}n+P_{\text{ten}},$$

where **P**_ten _is the cortical tension-driven rounding pressure defined in Eqn. 17. Using this formula, and a cell velocity of 10 *μ*m/min, we calculated the pressure profiles required to generate cell shapes seen in wild type cells as well as in *amiB*^- ^cells.

Obtaining these pressure profiles is straight-forward computationally, taking less than one minute of CPU time on a desk-top computer. It does require, however, a smooth shape. Thus, a certain amount of image processing is needed when using segmented images from experiments. Moreover, the formula in Eqn. 18 is based on a steady-state shape. Handling transient cell shape changes, such as protrusions or retractions, needs a local description of the velocity **v**(**x**).

Our results indicate that to generate polarized cell morphologies observed in wild type *Dictyostelium *cells, the protrusive forces must be primarily concentrated along the anterior ≈ 25% portion of the cell; see Fig. 7B. This is reminiscent of the PI(3,4,5)P_3 _threshold observed in cells [45,49]. At the sides, a smaller and less localized retractive force gives the cell its elongated shape. When this pressure profile was used to simulate a chemotaxing cell (Fig. 7C), the resulting virtual cell achieved an elongated shape and chemotaxed successfully to the source of chemoattractant achieving a stable velocity of 11.1 *μ*m/min.

Clearly, a different pressure profile is needed to generate a fan like shape as observed in *amiB*^- ^cells (Fig. 7D). Here, the maximum protrusive force is spread out considerably more at the front, while large amount of retraction force is still needed to pull the tail region along. Using this pressure profile in the chemotaxis simulation led to a migrating cell with stable shape similar to that seen experimentally (Fig. 7F). The resultant fan-shaped cell achieved the stable velocity of 9.7 *μ*m/min.

## Conclusion

We have shown that the simulation framework we have developed can be used to model cell shape deformations as well as cell motility. The simulations can produce deformations seen during micropipette aspiration experiments. This requires parameter values for the viscoelastic model which can be obtained experimentally. It should be noted, however, that 2-D simulations using parameters based on a 1-D model may not reproduce the 1-D model simulation precisely.

In the simulations of cell shape changes during chemotaxis, we saw that our simple model for generating the cell's protrusive and retractive forces in response to a chemoattractant gradient does not produce experimentally observed cell shapes. However, our techniques allow us to work backwards from shape to obtain the required forces. We determined that generating the elongated cell shape requires a large protrusive force at the front (the pressure profile there is positive). At the sides, there is a large retractive force (the pressure profile there is negative). While measuring this pressure profile directly would be difficult, it is possible to image fluorescently-tagged myosin-II to infer a measure of the forces acting on the cell. Under the assumption that the retractive force is being generated by myosin-II, we expect that myosin-II would be greatly enriched at the sides. Quantitatively, the spatial distribution of myosin could be used to estimate how much force is being generated along the membrane (as has been done during cytokinesis [50]).

## Authors' contributions

LY implemented the LSM simulations and drafted the manuscript. JCE performed experiments to measure the viscoelastic properties of cells, under the guidance of DNR. BLK and SES participated in the implementation of the LSM algorithm. PAI conceived of the study, and participated in its design and coordination. LY, JCE, DNR and PAI wrote the manuscript which was read and approved by all the authors.

## Competing interests

The authors declare that they have no competing interests.

## Supplementary Material

Additional file 1 This document presents detailed derivation of several of the formulae in the text. Click here for file
