# Supplementary material for: Modeling cellular deformations using the level set formalism
Source: BMC Syst Biol. 2008 Jul 24;2:68. doi: 10.1186/1752-0509-2-68 (PMC2535594; doi:10.1186/1752-0509-2-68)
Supplement: Additional file 1 — This document presents detailed derivation of several of the formulae in the text. [file 1752-0509-2-68-S1.pdf]

Supplementary Material

Derivation of $R_{\text{cap}}(L_p)$

When the cell is aspirated into the micropipette, we assume that the curve formed inside the micropipette is an arc of a sphere of radius R_{cap}. In this section, we derive the expression for R_{cap} given the cell radius R_c, pipette radius R_p, and length of protrusion L_p shown in Figure A1.

We start by noting that $\triangle ACF$ is a right-angle triangle. Therefore, the length of segment AC is: $|AC| = \sqrt{R_c^2 - R_p^2}$. Let X denote the length of segment CD; thus

$$X = |CD| = |AD| - |AC| = R_c - \sqrt{R_c^2 - R_p^2}.$$

Next we note that $\triangle BCF$ is also a right-angle triangle. Let Y represent the length of segment BC. Then:

$$R_{\text{cap}}^2 = Y^2 + R_p^2.$$

Given that $R_{\text{cap}} = X + Y + L_p$, we have:

$$(X + L_p)^2 + Y^2 + 2(X + L_p)Y = Y^2 + R_p^2, \quad \Longrightarrow \quad Y = \frac{R_p^2 - (X + L_P)^2}{2(X + L_p)}.$$

Finally,

$$\begin{aligned} R_{\text{cap}} &= X + Y + L_p \\ &= X + L_p + \frac{R_p^2 - (X + L_P)^2}{2(X + L_p)} \\ &= \frac{R_p^2 + (X + L_P)^2}{2(X + L_p)}. \end{aligned}$$

This formula applies while the center of the arc is outside the pipette ($Y \geq 0$). Thereafter, the radius of the cap equals the radius of the pipette ($R_{\text{cap}} = L_p$).

Derivation of effective velocity $\bar{\mathbf{v}}$

Given that the potential function ϕ has evolved from $\phi(t)$ to $\phi(t + \Delta t)$ in time Δt, we can find the speed of change in ϕ with respect to time:

$$\frac{\partial \phi}{\partial t} = \frac{\overline{\phi(t + \Delta t)} - \phi(t)}{\Delta t}.$$

Assuming that a velocity vector $\overline{\mathbf{v}}$ that is outward normal to the cell membrane is responsible for this evolution in ϕ, $\overline{\mathbf{v}}$ must satisfy the level set equation:

$$\frac{\partial \phi}{\partial t} + \overline{\mathbf{v}} \cdot \nabla \phi = 0.$$

Let $|\overline{\mathbf{v}}|$ represent the magnitude of vector $\overline{\mathbf{v}}$. Its direction is the normal vector:

$$\mathbf{n} = \frac{\nabla \phi}{|\nabla \phi|}.$$

This leads to:

$$\frac{\partial \phi}{\partial t} + |\overline{\mathbf{v}}| \frac{\nabla \phi}{|\nabla \phi|} \cdot \nabla \phi = 0.$$

Therefore,

$$\frac{\partial \phi}{\partial t} + |\overline{\mathbf{v}}| \frac{|\nabla \phi|^2}{|\nabla \phi|} = 0.$$

Solving for $|\overline{\mathbf{v}}|$, we have:

$$|\overline{\mathbf{v}}| = -\frac{1}{|\nabla \phi|} \frac{\partial \phi}{\partial t}.$$

Thus, incorporating the direction of the vector ($\mathbf{n}$) lead to the equation:

$$\overline{\mathbf{v}} = -\frac{\overline{\phi(t+\Delta t)} - \phi(t)}{\Delta t} \frac{\nabla \phi}{|\nabla \phi|^2}.$$

Fitting the pressure profiles generated from cell shape

In the main text we computed the local pressure profiles needed to generate steady-state elongated or fan-like cell shapes while chemotaxing. This leads to the discrete points of Figure 7B,E. However, to generate the profile during the simulations requires a continuous function of local chemoattractant concentration. This requires that the computed pressure profiles be smoothed and fitted by a curve.

First, we normalize the local chemoattractant (cAMP) concentration:

$$x = \frac{\text{cAMP} - \min(\text{cAMP})}{\max(\text{cAMP}) - \min(\text{cAMP})} - 0.5.$$

Thus, the normalized chemoattractant concentration ranges from -0.5 (at the rear) to $+0.5$ (at the front).

The computed profiles (red dots in Figure 7B,E) are now fitted to specific formulae. For the elongated cell, we found that the computed pressure profile can be accurately approximated by the ninth order polynomial:

$$y = a_1x^9 + a_2x^8 + a_3x^7 + a_4x^6 + a_5x^5 + a_6x^4 + a_7x^3 + a_8x^2 + a_9x + a_{10}.$$

Using a fitting program (Matlab's Curve Fitting Toolbox, The Mathworks, Natick, MA) to minimize the least squares error, we found the following values for the polynomial's coefficients:

$$\begin{aligned} a_1 &= -4318, & a_2 &= -489.1, \\ a_3 &= 1698, & a_4 &= 200.7, \\ a_5 &= -192.9, & a_6 &= -12.66, \\ a_7 &= 11.53, & a_8 &= 1.283, \\ a_9 &= 0.005968, & a_{10} &= -0.2812. \end{aligned}$$

For the fan shaped $amiB^-$ null cell, we found that the computed pressure profile could be approximated by a fifth order rational function:

$$y = \frac{n_1x^4 + n_2x^3 + n_3x^2 + n_4x + n_5}{x^5 + d_1x^4 + d_2x^3 + d_3x^2 + d_4x + d_5}.$$

Least square fit provided the following values for coefficients:

$$\begin{aligned} n_1 &= -12260, & d_1 &= -10340, \\ n_2 &= -4472, & d_2 &= -6104, \\ n_3 &= 3799, & d_3 &= 3391, \\ n_4 &= 2096, & d_4 &= 3062, \\ n_5 &= 262.5, & d_5 &= 615. \end{aligned}$$

The approximations are shown by the blue lines in Figure 7B,E.

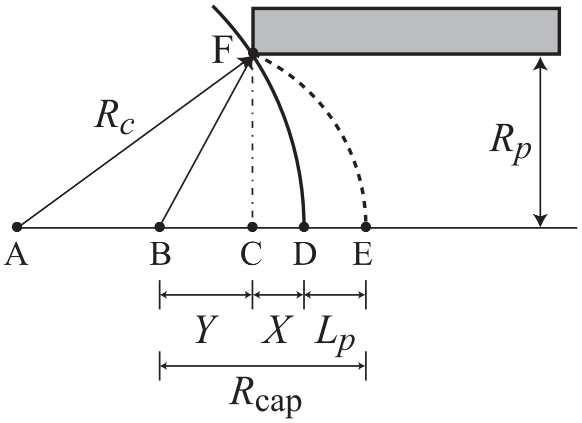

Supplementary Figure 1. Geometry of micropipette aspiration experiment. R_c is the initial radius of the spherical cell, R_p is the radius of the pipette and L_p is the measured protrusion into the pipette.
